# From arterial stiffness to kidney graft microvasculature: Mortality and graft survival within a cohort of 220 kidney transplant recipients

**DOI:** 10.1371/journal.pone.0195928

**Published:** 2018-05-03

**Authors:** Lynda Cheddani, Camélia Radulescu, Michel Chaignon, Alexandre Karras, Yann Neuzillet, Jean-Paul Duong, Nahid Tabibzadeh, Emmanuel Letavernier, Michel Delahousse, Jean-Philippe Haymann

**Affiliations:** 1 Service d’Explorations Fonctionnelles Multidisciplinaires, Assistance Publique—Hôpitaux de Paris (AP-HP), Hôpital Tenon, Paris, France; 2 Sorbonne Université, INSERM, UMR_S 1155, AP-HP, Hôpital Tenon, Paris, France; 3 Service d’Anatomopathologie, Hôpital Foch, Suresnes, France; 4 Service de Néphrologie, Assistance Publique—Hôpitaux de Paris (AP-HP), Hôpital Européen Georges-Pompidou, Paris, France; 5 Université Paris Descartes, Paris, France; 6 Institut National de la Santé et de la Recherche Médicale (INSERM) U970- PARCC, Paris, France; 7 Service d’Urologie, Hôpital Foch, Suresnes, France; 8 Service d’Anatomopathologie, Assistance Publique—Hôpitaux de Paris (AP-HP), Hôpital Necker, Paris, France; 9 Service de Néphrologie et Transplantation rénale, Hôpital Foch, Suresnes, France; 10 INSERM U-1018; CESP Team 5 (EpReC, Renal and Cardiovascular Epidemiology), Villejuif, France; University Jean MONNET of SAINT-ETIENNE, UNITED STATES

## Abstract

**Background:**

Aortic stiffness assessed by carotid-femoral pulse wave velocity (CF-PWV) is a predictor of mortality in several populations. However, little is known in kidney transplant recipients. Our objectives were to evaluate the ability of CF-PWV measured 3 months following transplantation to predict mortality, graft loss and its potential links to measured Glomerular Filtration Rate (mGFR) or kidney graft microvasculature parameters.

**Methods:**

The study is based on a monocentric retrospective cohort including 220 adult kidney graft recipients evaluated three months after transplantation. CF-PWV measures, clinical, laboratory and histological data performed at 3 (M3) and 12 months (M12) following transplantation were retrospectively collected. The two primary endpoints were all-cause mortality and occurrence of end stage renal disease (ESRD) defined by initiation of dialysis.

**Results:**

After a median follow up of 5.5 years [1.9; 8.8], death and graft loss occurred in 10 and 12 patients respectively. M3 CF-PWV was an independent mortality risk factor (HR = 1.29 [1.03; 1.61]; p = 0.03), despite no aortic stiffness variation during the first year of transplantation. Of notice, M3 CF-PWV was not associated with M12 mGFR or ESRD outcome. Graft microcirculation assessed by Banff vascular fibrous intimal thickening score (cv) worsened between M3 and M12 (p = 0.01), but no link was found with CF-PWV, mGFR or ESRD outcome. Surprisingly, acute rejections at M3 were associated after adjustment with mortality (p = 0.03) but not ESRD.

**Conclusion:**

Aortic stiffness measured 3 months after kidney transplantation is a strong predictor of mortality with no obvious influence on kidney graft microvasculature or graft loss.

## Introduction

Chronic kidney disease (CKD) dramatically increases risk of cardiovascular morbidity and mortality. If renal transplantation significantly improves patients survival in comparison to dialysis [1], cardiovascular (CV) death remains one of the main cause of graft loss during the first year following kidney transplantation [2]. Indeed, cardiovascular death annual risk still remains 50-fold higher in transplant recipients compared to general population [3]. In this population, as well as in patients with also end stage renal disease patients (ESRD), structural and functional modifications of large arteries are a striking feature, which may explain part of this important cardiovascular risk [4,5].

Carotid-femoral pulse wave velocity (CF-PWV) is a noninvasive and reproducible method currently considered as the gold standard for aortic stiffness measurement [6], and a marker of target organ damage in the European Society of Hypertension–European Society of Cardiology guidelines [7]. Indeed, arterial stiffness was reported as an independent predictive factor for coronary heart disease, fatal stroke, total and cardiovascular mortality in essential hypertensive [8–10], diabetic [11], and ESRD [12–14] populations.

However little is known about arterial stiffness in kidney transplant recipients. Indeed, vascular damages occurring during pre-transplantation era appear as one of the main cardiovascular risk factor [15]. However, renal transplantation may exert beneficial effects on aortic stiffness evolution through kidney function recovery [15]. Conversely, new-onset diabetes, hypertension and/or dyslipidemia may occur following renal grafting, due to immunosuppressive drug side effects [16,17]. Moreover, calcineurin inhibitors (CNI), which remain a cornerstone treatment in renal transplant patients, induce a renal and systemic vasoconstrictive effect leading to increased peripheral wave reflection [18]. Thus, it is difficult to extrapolate the results of studies carried out on ESRD patients to kidney transplant patients. Aortic stiffness and increased wave reflections were reported as independent predictors of cardiovascular events in renal transplant recipients [15]. To our knowledge, only one recent study reported CF-PWV measured at 8 weeks following grafting as a strong risk factor for mortality [19]. Thus the aim of our study was to investigate whether 3 months CF-PWV values can predict mortality but also graft loss after kidney transplantation. We also raised the issue whether 3 months CF-PWV value could influence renal function and kidney microvascular bed lesions as assessed by Banff vascular score 12 months after grafting.

## Materials and methods

### Study design

**Fig 1:**
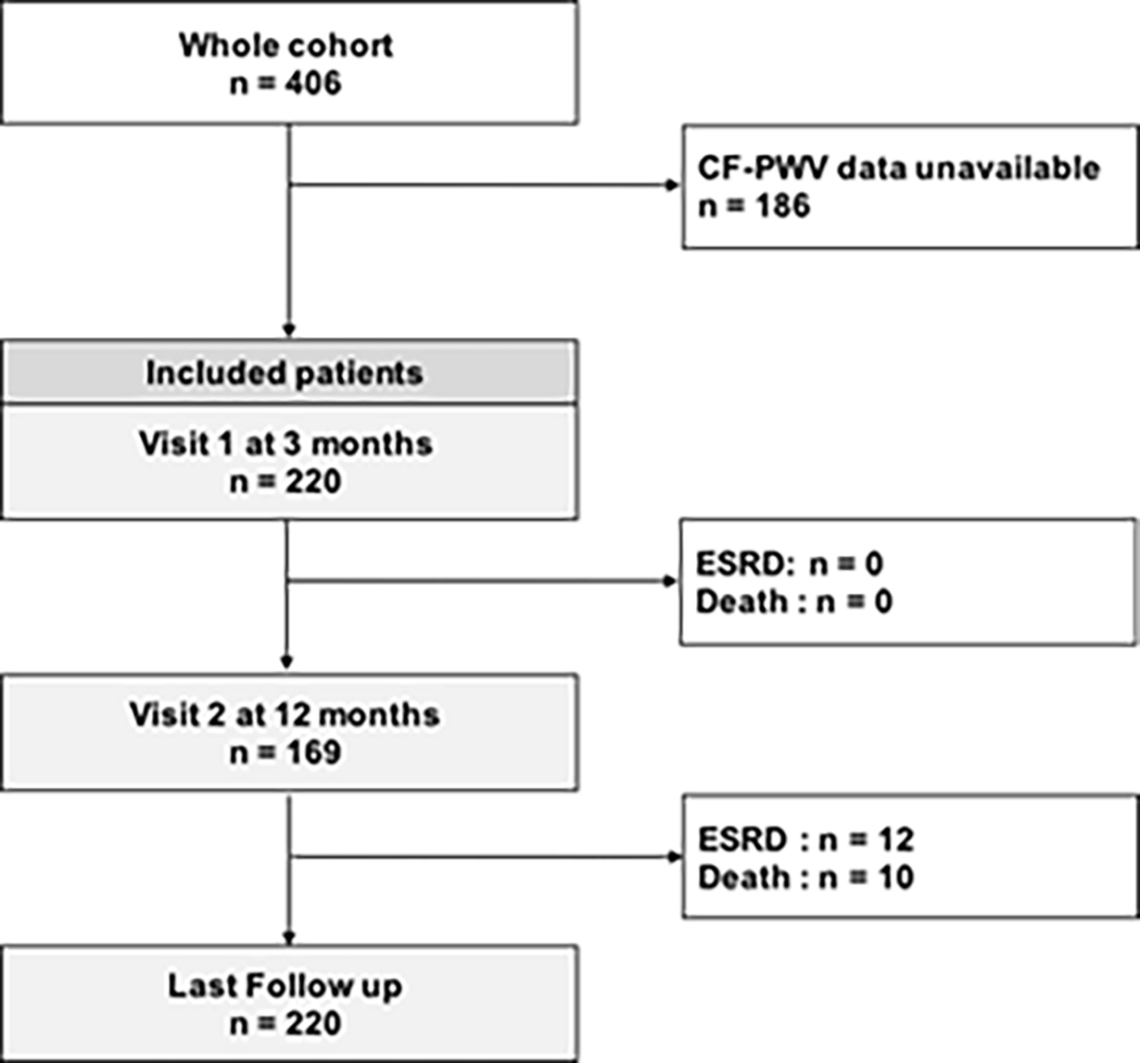
Study flow charts.

The present study is a monocentric retrospective hospital-based cohort which included adult kidney graft recipients evaluated between June 2007 and January 2016 in the department of renal Physiology (Tenon Hospital, Paris, France) three months after transplantation. Among the 406 recipients recorded, three months CF-PWV data were unavailable for 186 patients. Thus 220 patients were included in the study (Fig 1). Follow up was completed for all patients. The end of follow up was censored on January 1^st^, 2017.

All participants signed a written informed consent and data collection was approved by the CNIL according to French legislation (n° 2065902v0).

### Data and measurements

All kidney recipients received a standard immunosuppressive regimen including an induction therapy (methylprednisolone associated with basiliximab or Anti-thymocyte globulin) followed by a tritherapy associating CNI (tacrolimus or ciclosporin), corticosteroids (tapered to 5 mg/day after one month) and Mycophenolate mofetil.

Clinical and laboratory data at 3 (M3) and 12 months (M12) after kidney transplantation at the physiology department were retrospectively collected. They included demographics, medical history, primary renal disease diagnosis, height and weight, resting blood pressure (BP), and medications. Elevated blood pressure was defined by a systolic BP ≥140 mm Hg and/or diastolic BP ≥90 mm Hg and/or antihypertensive drugs intake. At each visits, GFR was measured by 51Cr-EDTA renal clearance as described previously [20]. In summary, 1.8 to 3.0 MBq of 51Cr-EDTA (GE Healthcare, Velizy, France) was injected intravenously as a single bolus. Average renal 51Cr-EDTA clearance was then determined over 5 to 6 consecutive 30-min clearance periods. Blood samples for creatinine measurements were obtained simultaneously. Serum creatinine was measured with an IDMS traceable enzymatic assay and GFR was estimated (eGFR) with creatinine-based MDRD equations [21]. Albumin-to-creatinine ratio (ACR) was measured in 60% of patients (133 out of 220 patients).

A protocol kidney graft biopsy was performed at M3 and M12 in n = 170 and 113 patients respectively (according to the benefit-risk ratio assessed individually). Renal graft biopsies were scored according to Banff classification by an experienced pathologist [22]. We collected peritubular capillaritis (ptc) and vascular fibrous intimal thickening (cv) parameters (scored between 0 and 3) in order to assess renal graft vascular damage.

### CF-PWV measurement

Patients were examined in a quiet, temperature controlled room, and measurements were performed by experienced operators. Blood pressure was measured after 15 min of rest in a supine position using a sphygmomanometer and a cuff of appropriate size. The average of five consecutive measurements was calculated. CF-PWV was measured along the descending thoraco-abdominal aorta using the foot-to-foot velocity method with an automatic device (Complior, Artech Medical, Pantin, France). This method enables online pulse wave recording and automatic calculation of CF-PWV by dividing the distance between carotid and femoral measurement sites by the transit time of the wave. Validation of this method and its reproducibility have been previously reported [23]. CF-PWV was defined as the mean of five determinations. Data concerning the validation of this method and its reproductibility have been previously described, with intraobserver and interobserver repeatability coefficients respectively of 0.94 and 0.89 [23].

### Outcomes

The two primary endpoints were all-cause mortality and occurrence of ESRD defined by initiation of dialysis. Events were identified from patients’ medical records or through record linkage with the French national registry of transplant recipients (Registre CRISTAL, Agence de Biomédecine). All survival data and graft survival data were right-censored on January 1st, 2017.

### Statistical analyses

In the overall population, we first used non-parametric Wilcoxon Mann-Withney or Fischer’s test as appropriate, to compare patients’ characteristics (at 3 and 12 months after grafting) according to the upper CF-PWV quartile. We then performed cause-specific Cox regression models to estimate crude and adjusted hazard ratios (HR) and their 95% confidence intervals (95% CI) for death associated with 3 months CF PWV, M3 mGFR or Banff vascular parameters. Adjustment covariates were similar in all analyses: recipient age, gender and diabetes. The proportional hazard assumption was checked for each model to ensure of their validity. Kaplan-Meier method was used to represent percentage survival as a function of the presence of the studied outcomes (death or ESRD). A log rank test was used to compare survival curves. All analyses and graphics were performed in R Statistical Software using the R-Studio interface (Version 3.3.2).

## Results

Among the 220 patients included in the study (i.e. evaluated 3 months after renal grafting), 169 (76.8%) repeated the evaluation at 12 months. After a median follow up of 5.5 years [1.9; 8.8], 12 patients underwent dialysis (5.45%) and overall mortality was reported for 10 patients (4.55%) and cardiovascular mortality for 7 patients (3.2%). Graft rejection was encountered in 11.2% of patients at M3. No patients were lost during follow up (Fig 1). Among the whole cohort, aortic stiffness (assessed by CF-PWV) and mGFR were similar between three and twelve months (mean = 9.5 versus 9.5 m/s, p = 0.95 and 57.1 versus 54.6 ml/min/1.73m^2^, p = 0.15, respectively) whereas renal microvessels (assessed by vascular fibrous intimal thickening score (cv)) significantly worsened (1.5 to 1.9, p = 0.01; n = 62).

As shown Tables 1 and 2, we compared the population according to M3 CF-PWV value below or above the upper CF-PWV quartile (10.6 m/s). Mean blood pressure or mGFR (and eGFR also, data not shown), did not differ between the two groups both at M3 and M12. In the higher CF-PWV group, patients were older (56.8 +/- 13.2 years vs 49.3 +/- 12.6 years, p < 0.001) with a higher prevalence of diabetes (43.9 vs 23.3%, p = 0.006) but did not differ for smoking (11.0% vs 8.8% p = NS). In this latter group, aspirin treatment was more frequent (p = 0.007), with older donors (56.1 vs 52.0%, p = 0.035), a longer cold ischemia time (995 min vs 756 min, p < 0.002), but no difference for Banff renal vessel score (i.e. vascular fibrous intimal thickening (cv) and peritubular capillaritis (ptc)) both at M3 and M12.

**Table 1:** Comparison of clinical and demographical characteristics at baseline according to M3 CF-PWV threshold of 10.6 m/s.

	Overall n = 220	CF-PWV < 10.6m/s n = 165	CF-PWV ≥ 10.6 m/s n = 55	p
***Recipient characteristics at baseline***				
Recipient age (years)	51.2 +/- 13.3	49.26 +/- 12.6	56.8+/-13.2	<0.001
Men (%)	62.8	54.6	71.9	0.027
BMI (kg/m^2^)	25.2 +/- 4.3	24.38 +/- 4.4	26.4 +/- 4.01	0.009
Obesity (%)	13.1	7.4	12.3	0.277
Smoking (%)	11.3	11.0	8.8	0.803
Diabetes (%)	33.0	23.3	43.9	0.006
NODAT (%)	19.0	19.6	17.5	0.846
Hypertension (%)	93.8	92.0	96.5	0.364
Statin (%)	35.0	34.4	38.6	0.630
Primary kidney disease (%)				0.006
Diabetic	6.4	2.5	17.5	
Glomerular	26.4	17.6	19.3	
Vascular	7.7	7.4	8.8	
Polycystic	21.4	22.8	17.5	
Other or unknown	38.2	38.9	36.8	
Preemptive Renal Transplantation (%)	12.3	13.0	10.5	0.815
Duration of dialysis (years)	2.8 +/- 3.2	2.9+/-3.5	2.5+/- 2.1	0.759
ACE Inhibitor and/or ARBs (%)	41.9	46.6	38.6	0.353
CCB (%)	57.6	51.5	71.9	0.008
Diuretics (%)	12.3	10.4	17.5	0.166
Beta Blockers (%)	44.1	36.8	52.6	0.042
Other antihypertensive drug (%)	15.5	11.0	17.5	0.248
Aspirin (%)	21.4	16.0	33.3	0.007
***Graft characteristics***				
Donor Age (years. median)	53.1 +/- 14.3	52.0+/-13.6	56.1 +/- 16.0	0.035
Deceased Donor (%)	75.4	71.2	82.5	0.115
Cause of death (%)				0.915
Anoxia	17.2	17.2	17.0	
Fatal cardiovascular event	56.4	54.3	61.7	
Traumatic	22.1	23.3	19.1	
Other	4.3	5.2	2.1	
Cold ischemia time (min)	818 +/- 534	756+/-515	995+/- 552	0.002
Acute rejection M3 (%)	11.2%	9.6%	16.2%	0.268

BMI: Body Mass Index; NODAT: New onset diabetes after transplantation; RRT: Renal Replacement Therapy; ACE: angiotensin-converting enzyme inhibitor; ARB: angiotensin receptor blocker; CCB: calcium channel blocker.

**Table 2:** Comparison of arterial and renal parameters according to M3 CF-PWV threshold of 10.6 m/s.

	Overall n = 220	CF-PWV < 10.6m/s n = 165	CF-PWV ≥ 10.6 m/s n = 55	p
***Arterial Parameters***				
MBP M3 (mmHg)	95.0 +/- 12.0	95.2 +/- 12.6	94.6 +/- 10.3	0.927
MBP M12 (mmHg)	95.0 +/- 12.0	92.7 +/- 11.6	92.5 +/- 11.0	0.778
PP M3 (mmHg)	57.0 +/- 12.7	54.0+/- 13.0	60 +/- 11.5	0.011
PP M12 (mmHg)	56.0 +/- 12.7	53.7 +/- 12.0	62.7 +/- 12.2	<0.001
HR M3 (bpm)	79.0 +/- 13.4	78 +/- 12.1	76 +/- 12.0	0.665
HR M12 (bpm)	77.9 +/- 12.1	77.8 +/- 12.1	78.4 +/- 12.0	0.665
CF-PWV M3 (m/s)	9.5 +/- 2.3	8.49 +/- 1.2	12.4 +/- 2.2	<0.001
CF-PWV M12 (m/s)	9.3 +/- 1.9	8.68 +/- 1.7	11.5 +/- 2.4	<0.001
Delta CF-PWV (M12 –M3)	-0.08 +/- 1.5	+0.11 +/- 1.4	-0.52 +/- 1.77	0.007
Delta MAP (M12—M3)	-2.54+/-12.4	-2.98 +/- 12.8	-1.24 +/- 11.3	0.306
***Renal Parameters***			
Creatinine M3 (μmol/L)	118.0+/- 42.4	117 +/- 38.5	121 +/- 52.1	0.983
mGFR M3	57.1 +/- 16.6	57.5 +/- 15.7	56.0 +/- 19.1	0.440
mGFR M12	54.6 +/- 17.9	54.5 +/- 16.1	54.8 +/- 22.7	0.811
Delta mGFR (M12 –M3)	-2.68 +/- 12.04	-2.93 +/- 11.9	-1.90 +/- 12.6	0.540
mGFR improvement ≥ 5 ml/mn/1,73m^2^	24.2	20.6	26.9	0.343
Banff cv M3	1.5 +/- 0.7	1.5 +/- 0.7	1.5 +/- 0.7	0.915
Banff cv M12	1.8 +/- 0.9	1.8 +/- 0.8	2.0 +/- 1.2	0.675
Delta cv (M12-M3)	0.27 +/- 0.9	0.31 +/- 0.9	0.17 +/- 1.0	0.411
ptc Banff M3	1.2 +/- 0.5	1.2 +/- 0.4	1.3 +/- 0.6	0.678
ptc Banff M12	1.4 +/- 0.7	1.3 +/- 0.6	1.6 +/- 0.9	0.476
Delta ptc (M12-M3)	0.1 +/- 0.7	0.1 +/- 0.5	0.2 +/- 1.0	0.906
ACR M3 (mg/mmol)	14.0+/- 45.0	11.2 +/- 37.0	9.35 +/- 9.2	0.041
Microalbuminuria M3 (%)	46.2	41.8	38.9	1
Macroalbuminuria M3 (%)	8.3	10.1	11.1	1

MBP: Mean Blood Pressure; PP: Pulse Pressure; HR: Heart Rate; mGFR: measured Glomerular Filtration Rate; Banff cv: vascular fibrous intimal thickening score; Banff ptc: peritubular capillaritis score; ACR: albumin creatinine ratio.

Among high M3 CF-PWV patients (>10.6 m/s), a significant CF-PWV improvement was noticed between M3 and M12 (-0.5 m/s, p = 0.03), despite a similar MBP and no significant cv score or mGFR variation (p = 0.61 and 0.42 respectively). Conversely, in the lower M3 CF-PWV group, no CF-PWV improvement was noticed (+0.11 m/s, p = 0.63) though cv score worsened (from 1.5 to 2.0, p = 0.03).

**Fig 2:**
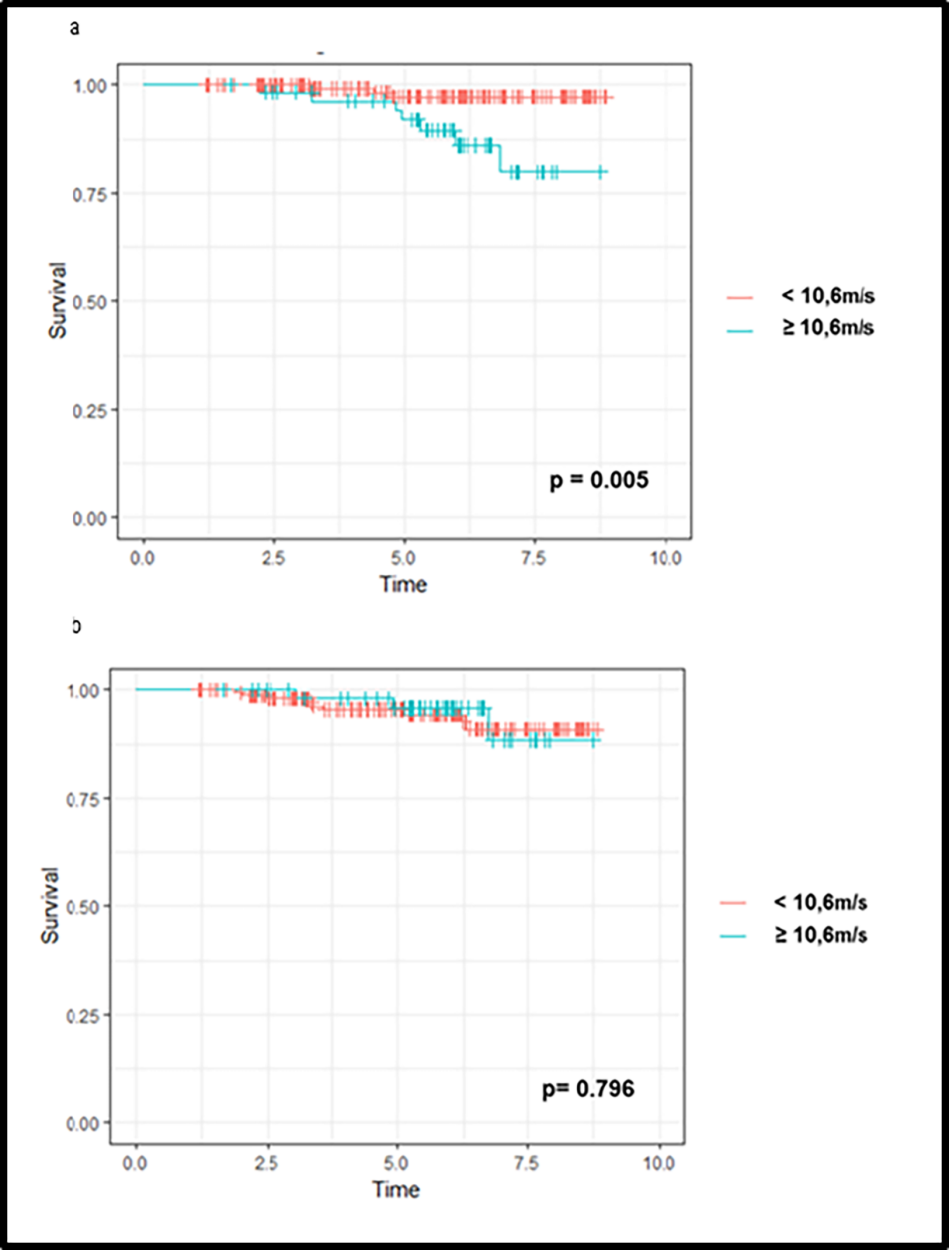
Unadjusted Kaplan-Meier event curves for all-cause of mortality (a) or kidney graft loss (b) according to M3 CF-PWV threshold of 10.6 m/s.

As shown Fig 2A, mortality was significantly higher in patients with M3 CF-PWV >10.6 m/s (p = 0.001). Univariate Cox analysis (Table 3) shows that mortality was associated with M3 CF-PWV (HR: 1.38 [1.18; 1.62], p < 0.001), but also recipient age (HR: 1.10 [1.03;1.16], p = 0.003), diabetic status (HR: 2,98 [1.28;6.92], p = 0.01), number of antihypertensive drugs (HR: 1.82 [1.07;3.09], p = 0.03), cv Banff score at 3 months (HR: 2.92 [1.39; 6.15], p = 0.005), M3 mGFR (HR: 0.97 [0.94;0.99], p = 0.01) and acute rejection both at M3 and M12 (HR: 8.3 [2.2; 30.9], p = 0.002 and HR: 6.6 [1.1; 40.0], p = 0.04 respectively). Interestingly, mortality was associated with M12 CF-PWV (HR: 1.34 [1.10; 1.64], p = 0.004), but not aortic stiffness change between 3 and 12 months (p = 0.70).

**Table 3:** Crude hazard ratios (95% IC) of death and ESRD.

	All cause mortality		ESRD	
	HR (95% CI)	p	HR (95% CI)	p
***Recipient characteristics Baseline***				
***Demographics***				
Recipient age (years. median)	1.10 [1.03;1.16]	0.003	0.98 [0.95;1.02]	0.386
Men (%)	0.47 [0.13;1.66]	0.241	1.42 [0.43;4.73]	0.564
BMI (kg/m2)	1.04 [0.96;1.12]	0.391	1.03 [0.93;1.14]	0.531
Obesity (%)	2.38 [0.56;11.23]	0.272	0.92 [0.12;7.12]	0.936
Smoking (%)	0.98 [0.12;7.75]	0.986	1.59 [0.36;7.09]	0.543
***Clinics***				
Diabetes (%)	2.98 [1.28;6.92]	0.011	1.23 [0.42;3.61]	0.705
NODAT (%)	1.04 [0.22;4.88]	0.965	1.80 [0.57;5.65]	0.316
Statin treatment (%)	1.86 [0.80;4.3]	0.148	0.86 [0.26;2.86]	0.803
Preemptive Renal Transplantation (%)	0.86 [0.11;6.82]	0.890	NS	0.998
Duration of dialysis (year)	0.99 [0.89;1.11]	0.869	1.05 [0.94;1.17]	0.377
***Immunology***				
PRA (%)	1.00 [0.97;1.04]	0.920	1.00 [0.98;1.03]	0.950
HLA Class I Antibodies (%)	0.93 [0.75;1.15]	0.494	1.01 [0.99;1.04]	0.355
HLA Class II Antibodies (%)	0.99 [0.94;1.04]	0.648	0.99 [0.95;1.03]	0.742
HLA missmatch	0.93 [0.65;1.32]	0.689	1.11 [0.80;1.54]	0.527
***Medication use***				
ACE Inhibitor or ARBs (%)	1.54 [0.43;5.47]	0.504	0.34 [0.11;1.08]	0.068
Calcium Channels Blockers (%)	0.99 [0.28;3.52]	0.992	1.35 [0.41;4.47]	0.627
Diuretics (%)	4.11 [1.16;14.56]	0.029	2.17 [0.59;8.02]	0.246
Beta Blockers (%)	3.25 [0.84;12.57]	0.088	0.72 [0.22;2.39]	0.589
Number of antihypertensive drugs	1.82 [1.07;3.09]	0.027	0.97 [0.57;1.65]	0.918
Aspirin (%)	2.86 [0.80;10.17]	0.105	1.45 [0.39;5.39]	0.579
***Arterial Parameters***				
SBP M3 (mmHg)	1.00 [0.96;1.04]	0.914	0.99 [0.96;1.03]	0.739
DBP M3 (mmHg)	0.94 [0.89;0.99]	0.029	0.99 [0.94;1.04]	0.762
MBP M3(mmHg)	0.97 [0.93;1.00]	0.071	0.99 [0.94;1.04]	0.723
PP M3 (mmHg)	1.05 [1.01;1.10]	0.046	0.99 [0.95;1.04]	0.893
HR M3 (bpm)	0.95 [0.90;1.01]	0.086	1.01 [0.97;1.05]	0.754
CF-PWV M3 (m/s)	1.38 [1.18; 1.62]	< 0.001	0.85 [0.62;1.15]	0.285
CF-PWV M12 (m/s)	1.34 [1.10; 1.64]	0.004	0.84 [0.60;1.17]	0.306
**Delta CF-PWV (M12-M3)**	1.09 [0.71.1.76]	0.696	0.85 [0.56.1.29]	0.435
***Renal Parameters***				
Creatinine M3 (μmol/L)	1.00 [0.99;1.02]	0.689	0.95 [0.92;0.99]	0.004
mGFR M3 (ml/mn/1.73m^2^)	0.97 [0.94;0.99]	0.014	0.95 [0.92;0.99]	0.007
Measured Creatinine Clearance M3 (ml/mn/1.73m^2^)	0.99 [0.96;1.02]	0.531	0.94 [0.91;0.98]	0.002
eGFR M3 (ml/mn/1.73m^2^)	0.98 [0.95;1.01]	0.221	0.94 [0.91;0.98]	0.002
mGFR M3 < 30 ml/mn/1.73m^2^ (%)	4.27 [0.53;34.09]	0.171	7.84 [1.71;36.07]	0.008
Delta mGFR (M12 –M3)	0.55 [0.14;2.12]	0.381	0.65 [0.20;2.16]	0.481
***Albuminuria M3 (n = 133)***				
Log(ACR)	1.059 [0.56 ;1.99]	0.859	1.39 [0.85 ; 2.27]	0.190
Albuminuria > 3 mg/mmol (%)	0.75 [0.11 ;5.34]	0.776	1.31 [0.31 ;5.47]	0.715
Microalbuminuria (%)	0.93 [0.13 ;6.57]	0.938	1.09 [0.27 ; 4.36]	0.903
Macroalbuminuria (%)	3^e-8^ [0 ; Inf]	0.938	1.87 [0.23 ;15.42]	0.559
***Graft characteristics***				
Donor Age (year)	1.05 [1.00;1.10]	0.052	1.01 [0.97;1.05]	0.717
Deceased Donor (%)	1.19 [0.25;5.64]	0.824	1.48 [0.32;6.79]	0.614
Cold ischemia time (min)	1.00 [0.99;1.00]	0.439	1.15 [0.32;4.10]	0.827
Acute rejection M3 (%)	8.30 [2.23;30.92]	0.002	1.00 [0.13 ;8.13]	1
Acute rejection M12 (%)	6.63 [1.10;39.81]	0.039	1.73 [0.21;14.44]	0.612
Banff CV M3 (n = 135)	2.92 [1.39 ; 6.15]	0.005	0.55 [0.15;2.03]	0.369
Delta Banff cv (M12 –M3) (n = 86)	0.96 [0.35;2.67]	0.945	1.18 [0.49;2.84]	0.712
Banff ptc M3 (n = 134)	1.59 [0.59 ; 4.28]	0.359	0.56 [0.08;3.72]	0.546
Delta Banff ptc (M12 –M3) (n = 88)	3.93 [1.57.9.80]	0.003	1.80 [0.61; 5.24]	0.285

ACE: angiotensin-converting enzyme inhibitor; ACR: albumin creatinine ratio; ARB: angiotensin receptor blocker; BMI: Body Mass Index; CCB: calcium channel blocker; NODAT: New onset diabetes after transplantation; mGFR: measured Glomerular Filtration Rate; PRA: panel reactive antibody; RRT: Renal Replacement Therapy, MBP: Mean Blood Pressure, PP: Pulse Pressure, SBP: Systolic Blood Pressure, DBP: Diastolic Blood Pressure, Banff CV: vascular fibrous intimal thickening score, Banff ptc: peritubular capillaritis score.

However, as shown Table 4, M3 CF-PWV (and not M3 mGFR value or M3 Banff renal vessel score) was the only significant determinant of mortality after adjustment for conventional mortality risk factors (Model 1; HR: 1.25 [1.02; 1.55], p = 0.036). Surprisingly, acute rejection at M3 was also significantly associated with mortality (about 4.5 fold increase) after adjustment to other mortality risk factors (see model 4).

**Table 4:** Adjusted HRs (95% IC) of all-cause mortality associated with M3 CF-PWV, M3 mGFR, Banff vascular parameters and rejection episode.

	All cause mortality	
	Model 1	
	HR (95% CI)	p
Recipient age (year)	1.05 [0.97; 1.14]	0.215
Gender	0.64 [0.16; 2.63]	0.537
Diabetes	4.82 [0.94; 24.80]	0.060
PP (mmHg)	1.04 [0.98; 1.10]	0.189
CF-PWV M3 (m/s)	1.25 [1.02; 1.55]	0.036
	R^2^ = 0.11, Proportional hazards assumption: Respected	
	Model 2	
	HR (95% CI)	p
Recipient age (year)	1.08 [1.01; 1.16]	0.032
Gender	0.54 [0.14; 2.15]	0.386
Diabetes	5.59 [1.14; 27.37]	0.033
PP (mmHg)	1.03 [0.98; 1.09]	0.254
mGFR M3	0.97 [0.94; 1.01]	0.146
	R^2^ = 0.10, Proportional hazards assumption: Respected	
	Model 3	
	HR (95% CI)	p
Recipient age (year)	1.07 [0.97 ;1.18]	0.160
Gender	0.85 [0.19 ; 3.78]	0.834
Diabetes	3.82 [0.69 ; 21.26]	0.126
PP (mmHg)	0.94 [1.01 ;1.14]	0.027
Banff cv score M3	2.44 [0.98.6.09]	0.060
	R^2^ = 0.15, Proportional hazards assumption: Not Respected	
	Model 4	
	HR (95% CI)	p
Recipient age (year)	0.07 [0.98 ; 1.17]	0.088
Gender	0.58 [0.13 ; 2.56]	0.472
Diabetes	4.57 [0.87 ;23.96]	0.072
PP (mmHg)	1.05 [1.00 ; 1.11]	0.061
Acute rejection M3	4.49 [1.12 ;17.97]	0.034
	R^2^ = 0.16, Proportional hazards assumption: Respected	

mGFR: measured Glomerular Filtration Rate; PP: Pulse Pressure; cv Banff score: vascular fibrous intimal thickening.

In contrast to mortality, graft survival was similar between M3 CF PWV groups (Fig 2B). Indeed, univariate Cox analysis shows that kidney graft loss was only associated with M3 mGFR (HR: 0.95 [0.92;0.99], p = 0.007) but not M3 CF-PWV (HR: 0.85 [0,62;1.15], p = 0.28) or vascular Banff score (Table 3).

## Discussion

Our results show that M3 CF-PWV value is an independent risk factor for mortality with no influence on renal outcome.

Our results are consistent with one previous study reporting in a large cohort of kidney transplant recipients (4.2 years median follow up), that a high 8-week CF-PWV value (above 12 m/s) was indeed a mortality risk factor [19]. However aortic stiffness evolution during follow up was not available and graft survival was not evaluated in this latter study. Aortic stiffness evolution is presumably a slow process [24] and graft influence (i.e. donor characteristics) is probably mild on CF-PWV value 3 months after grafting. Indeed, in a previous study, we showed that CF-PWV values were independently associated with donor age at M12 but not at M3 [5]. However, other factors such as cold ischemia time may influence M3 CF-PWV (see Table 2).

When studying the whole population, a striking finding is the fact that M3 CF-PWV value (but also M12 CF PWV) was a mortality risk factor despite no significant aortic stiffness change during the first year of kidney transplantation. Thus transplantation would exert a protective effect on aortic stiffening compared to dialysis [25] but obviously could not strikingly improve aortic compliance to such an extent that mortality risk factor could be offset. Nevertheless, a significant aortic stiffness improvement was noticed in the high CF PWV group between M3 and M12 (p = 0.02). This beneficial effect though interesting remained however mild with an improvement above 1 m/s in only 16.3% of patients (our cohort is unfortunately too small to detect an impact on mortality in this subgroup).

Of notice, the finding that M3 CF-PWV was associated with mortality (including cardiovascular mortality as shown in S1 Fig and S1 Table) and not ESRD, suggests that pulsatile stress at M3 would be deleterious on transplanted recipient heart and vessels (i.e. target organ damage) but only to a lesser extent on renal vessels including renal microcirculation. We thus speculate that a preserved renal hemodynamic response is at play (i.e. an efficient myogenic response characterized by vasoconstriction and increased vascular resistance) ensuring a protecting downstream glomerular and peritubular microvessels, thus explaining that M3 CF-PWV (conversely to mGFR at M3) is indeed not a risk factor for ESRD (see Table 3).

Last, our finding that acute rejection during the first 3 months of transplantation was associated with mortality (by a 4.5 fold increase) is puzzling and should deserve confirmation in further studies (drug toxicity? immunologic factors?). Nevertheless, our data showing a significant association between acute rejection and M12 mGFR decrease and not ESRD outcome, confirm and extend a previous report showing in living donor renal transplanted patients that rejection was associated only with the first year eGFR decline and not after (at the end of the follow up) [26].

## Strength / Limitations / Confounders

This study had several strengths. It is the first study to combine two gold standard methods: aortic stiffness assessed by CF-PWV and mGFR measured with ^51^Cr-EDTA urinary clearance technique. Despite a relatively small cohort size thus explaining few death and graft loss events, our results demonstrate a significant link between a noninvasive aortic stiffness measurement and a hard clinical endpoint: mortality. Cardiovascular mortality was specifically addressed in S1 Fig and S1 Table and provides similar results as all-cause mortality results, though the number of events is indeed low (n = 7). Histopathological evaluation both at 3 and 12 months after kidney graft transplantation was available only in 69 patients and thus could account for unexpected bias or loss of statistical power. Nevertheless, the renal endothelial lesions assessed by the worsening of Banff cv score during the first year of transplantation are original findings that should deserve further studies aiming at identifying the main relevant determinants.

## Conclusions and perspectives

Three months CF PWV is a strong predictor of mortality with no obvious influence on renal outcome or kidney graft microvasculature, thus suggesting an efficient hemodynamic graft autoregulation at least in most patients. Slow aortic stiffness evolution reported for most patients, strengthens the view that transplantation, in comparison to dialysis, ensures a significant cardio vascular protection during the first year, and in some cases a reversal of arterial maladaptative remodeling [27], despite the fact that all risk factors are not completely offset. Performing M3 CF PWV measurements thus appears as an interesting tool after renal transplantation in order to identify and better monitor high cardiovascular risk patients.

## Supporting information

S1 Dataset (XLSX)

S1 Fig Unadjusted Kaplan-Meier event curves for cardio-vascular mortality according to M3 CF-PWV threshold of 10.6 m/s. (PNG)

S1 Table Crude hazard ratios (95% IC) of cardio-vascular death. ACE: angiotensin-converting enzyme inhibitor, ACR: albumin creatinine ratio, ARB: angiotensin receptor blocker, BMI: Body Mass Index, CCB: calcium channel blocker, NODAT: New onset diabetes after transplantation, mGFR: measured Glomerular Filtration Rate, PRA: panel reactive antibody, MBP: Mean Blood Pressure, PP: Pulse Pressure, SBP: Systolic Blood Pressure, DBP: Diastolic Blood Pressure, Banff CV: vascular fibrous intimal thickening score, Banff ptc: peritubular capillaritis score.
(PNG)

## Abbreviations

51Cr- EDTA: 51Cr-ethylenediaminetetraacetate (EDTA)
ACE Inhibitor: Angiotensin converting enzyme inhibitors
ARBs: Angiotensin receptor blockers
BMI: Body Mass Index
CCB: calcium channel blocker
CF-PWV: Carotido-femoral Pulse Wave Velocity
CKD: Chronic Kidney Disease
CNI: calcineurin inhibitor
CNIL: Commission Nationale de l'Informatique et des Libertés
cv: vascular fibrous intimal thickening
DBP: Diastolic Blood Pressure
eGFR: estimated Glomerular Filtration Rate (MDRD)
ESRD: End Stage Renal Disease
HR: Hazard ratio
M3: evaluation at 3 months
M12: evaluation at 12 months
MBP: Mean Blood Pressure
mGFR: Measured Glomerular Filtration Rate
NODAT: New Onset Diabetes after Transplantation
PP: Pulse Pressure
PRA: Panel Reactive Antibody
ptc: peritubular capillaritis
SBP: Systolic Blood Pressure
