# Supplementary figures and images for: From arterial stiffness to kidney graft microvasculature: Mortality and graft survival within a cohort of 220 kidney transplant recipients
Source: PLoS One. 2018 May 3;13(5):e0195928. doi: 10.1371/journal.pone.0195928 (PMC5933694; doi:10.1371/journal.pone.0195928)

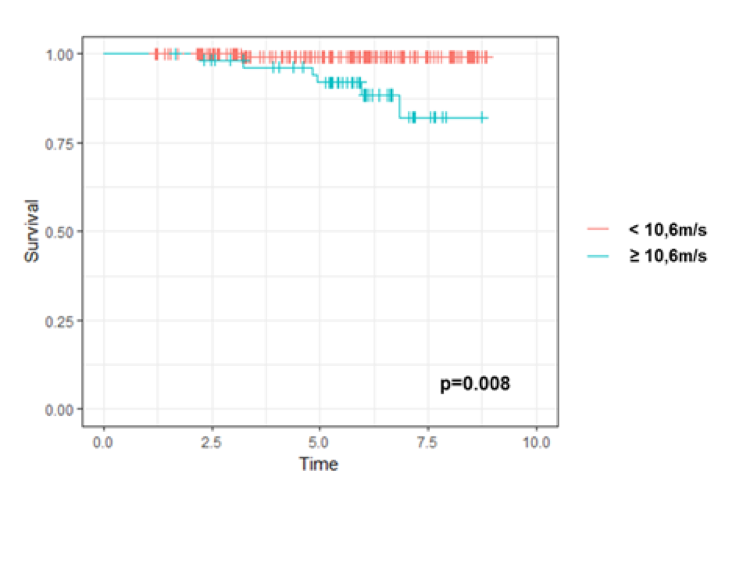

Supplement: S1 Fig — (PNG) [file pone.0195928.s002.png]

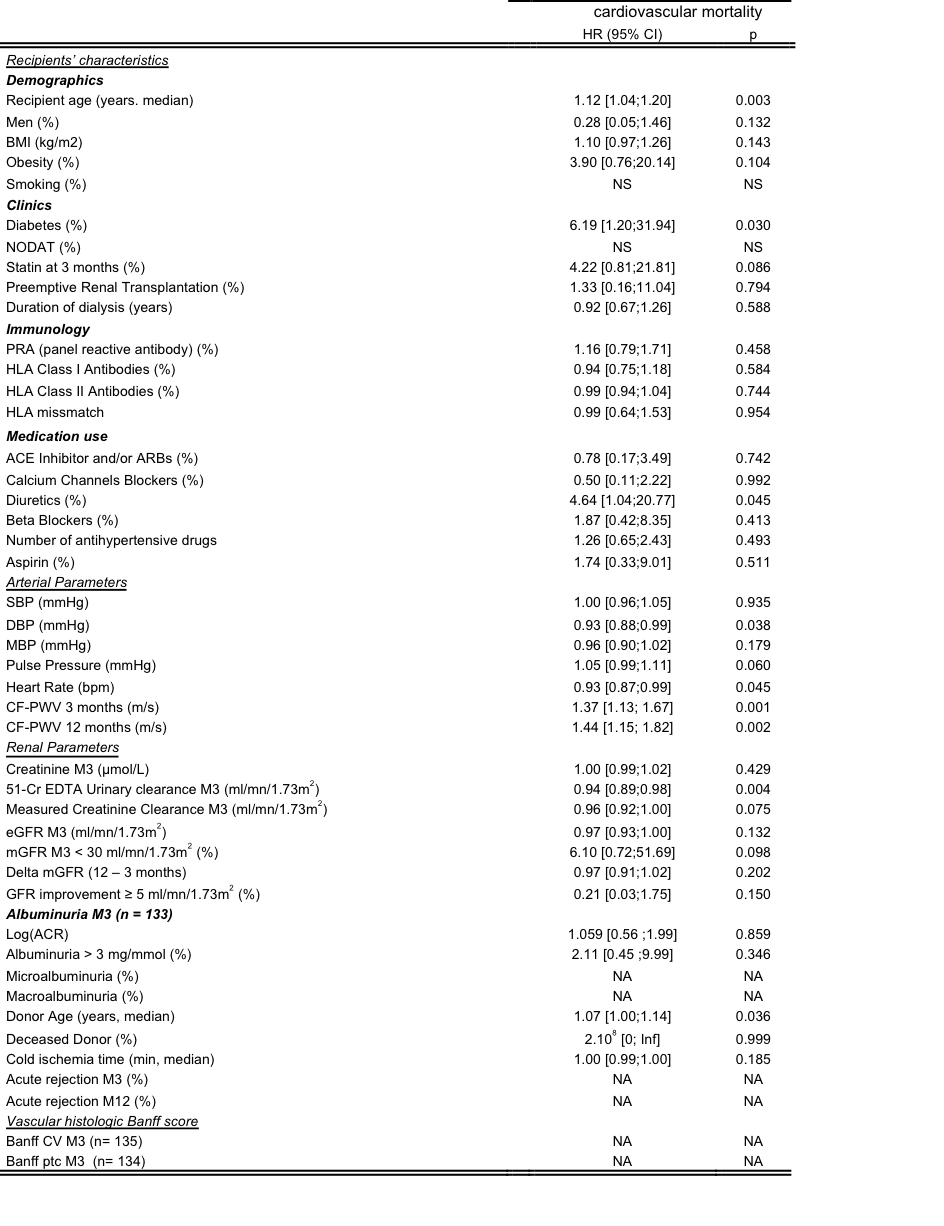

Supplement: S1 Table — ACE: angiotensin-converting enzyme inhibitor, ACR: albumin creatinine ratio, ARB: angiotensin receptor blocker, BMI: Body Mass Index, CCB: calcium channel blocker, NODAT: New onset diabetes after transplantation, mGFR: measured Glomerular Filtration Rate, PRA: panel reactive antibody, MBP: Mean Blood Pressure, PP: Pulse Pressure, SBP: Systolic Blood Pressure, DBP: Diastolic Blood Pressure, Banff CV: vascular fibrous intimal thickening score, Banff ptc: peritubular capillaritis score. (PNG) [file pone.0195928.s003.png]
